# Using Normalisation Process Theory to explore the contribution of stakeholder workshops to the development and refinement of a complex behavioural intervention: the STAMINA lifestyle intervention

**DOI:** 10.1186/s43058-024-00629-1

**Published:** 2024-09-02

**Authors:** Sophie Reale, Rebecca R. Turner, Liz Steed, Steph J. C. Taylor, Derek J. Rosario, Liam Bourke, Dylan Morrissey, Aidan Q. Innes, Eileen Sutton

**Affiliations:** 1 Allied Health Professions, College of Health, Wellbeing & Life Sciences, Sheffield Hallam University, Sheffield, UK; 2 Division of Medical Education, Faculty of Biology, Medicine and Health, University of Manchester, Manchester, UK; 3 Wolfson Institute of Population Health, Queen Mary University of London, London, UK; 4 Sheffield Teaching Hospitals NHS Foundation Trust, Department of Urology, Royal Hallamshire Hospital, Sheffield, S10 2JF UK; 5 Sport and Exercise Medicine, Queen Mary University of London, London, UK; 6 Physiotherapy Department, Barts Health NHS Trust, London, UK; 7 Nuffield Health, 2 Ashley Avenue, Epsom, Surrey UK; 8 British Population Health Sciences Institute, University of Bristol, Bristol, UK

**Keywords:** Intervention development, Normalisation process theory, Behaviour change, Prostate cancer, Exercise, Lifestyle, Stakeholders, Complex intervention, Process evaluation, Implementation

## Abstract

**Background:**

The National Institute for Health and Care Excellence (NICE) recommend that men with prostate cancer on androgen deprivation therapy (ADT) are offered twice weekly supervised aerobic and resistance exercise to address side effects of treatment. However, supervised exercise is not routinely offered in standard clinical practice. The STAMINA programme grant for applied research (PGfAR) has been designed to evaluate whether this recommendation can be delivered within standard NHS care. This paper describes how future implementation of NICE recommendations within the NHS was explored during complex intervention development to enable evaluation of a lifestyle intervention.

**Methods:**

Two stakeholder workshops were conducted to explore factors pertinent to future implementation of the STAMINA Lifestyle intervention (SLI). Normalisation Process Theory (NPT) provided the theoretical framework for discussion and analysis. Stakeholder workshop 1 focussed on intervention coherence and buy-in. Stakeholder workshop 2 explored barriers and facilitators for embedding SLI into the context of the NHS, with delivery partner Nuffield Health, in the future.

**Results:**

Workshops were attended by healthcare professionals (*n* = 16), exercise professionals (*n* = 17), members of public involved in PPI including patients (*n* = 12), health psychologists (*n* = 2), clinical commissioners (*n* = 4), cancer charities (*n* = 3), a cancer alliance (*n* = 1) and health economist (*n* = 1). Stakeholders agreed that professional training packages should emphasise the uniqueness of the SLI and underpinning theory and evidence (*Coherence*). To further engagement, the use of STAMINA champions and information about the delivery partner were recommended to enhance confidence and knowledge (*Cognitive participation*). Furthermore, a simple communication (*Collective Action*) and progress reporting system (*Reflexive Monitoring*) was suggested to fit into existing infrastructure within the NHS and community partner.

**Conclusions:**

Application of NPT within two stakeholder workshops enhanced complex intervention development. Context-specific strategies to support implementation of SLI within the context of a trial were proposed, sensed-checked, and considered acceptable. The organisational implications of embedding and sustaining the intervention in preparation for wider NHS roll-out were considered (if proven to be effective) and will be explored in the qualitative component of a process evaluation underpinned by NPT.

**Trial registration:**

(ISRCTN: 46385239). Registered on July 30, 2020.

**Supplementary Information:**

The online version contains supplementary material available at 10.1186/s43058-024-00629-1.

Contributions to the literature

- It takes on average 17 years for 14% of original research to be integrated into clinical practice.
- We demonstrate how implementation science and behavioural science theories and frameworks can be applied in parallel to optimise intervention development that might contribute to future implementation of complex interventions into the NHS and charity sector (i.e., Nuffield Health), in a time and resource efficient way.
- These findings address recognised gaps between research, policy, and practice by identifying acceptable context specific implementation facilitators to support the embedding of supervised exercise into standard NHS prostate cancer care (if found to be clinically and cost effective).

## Background

Supervised exercise is a safe and clinically efficacious solution to address the adverse effects of androgen deprivation therapy (ADT) for prostate cancer [1]. Clinically meaningful improvements in fatigue and cancer specific quality of life are demonstrated following twelve weeks of supervised exercise and dietary support, with improvements being sustained up to 6-month follow-up [1, 2]. As such the National Institute for Health and Care Excellence (NICE; NG131 1.4.19) recommend that men with locally advanced and advanced prostate cancer on ADT should be offered supervised, aerobic and resistance exercise at least twice a week for 12 weeks [3]. However, very few National Health Service (NHS) trusts have established adequate provision for such supervised services according to this recommendation [4]. This deficit is emblematic of the barriers to translating and embedding high-quality evidence and national recommendations into complex healthcare systems.

At present, it is estimated that less than half of all novel clinical innovations with proven efficacy are translated into routine healthcare [5]. Furthermore, it takes a considerable length of time for research findings to influence policy and practice [6]. Fortunately, significant recent advances in Implementation Science have yielded theories and frameworks that guide researchers towards embedding and evaluating interventions in real-world settings [7]. As such, it is recommended that factors pertinent to the immediate and future implementation of interventions (i.e., within a trial and healthcare setting respectively) are explored early, during complex intervention development. Failure to consider implementation during intervention development and subsequent trial evaluation may slow or halt the uptake and translation of proven healthcare interventions into future routine care.

Normalisation Process Theory (NPT) [8] was developed to understand and overcome difficulties encountered when implementing complex behavioural interventions into healthcare practice. Recent guidance on intervention development and evaluation stress the importance of encompassing strong theoretical foundations and engaging diverse stakeholders at all stages [9, 10]. NPT is a mid-range theory that can be applied at different stages of research to provide an explanatory framework to help understand how social practices are or may be implemented, embedded, and integrated into routine practice [11]. This contrasts to other frameworks such as the Theoretical Domains Framework (TDF) which take a more (though not solely) individual perspective on behaviour change and were used in this programme of work during earlier parts of the intervention development [12, 13]. NPT suggests the implementation of an intervention is operationalised through four main constructs: understanding the way people make sense of the work of implementing and integrating an intervention individually and as a team (construct 1: coherence); how they engage with that intervention (construct 2: cognitive participation); how they enact it in day-to-day practice (construct 3: collective action); and how they appraise its effects (construct 4: reflexive monitoring).

In this paper, we discuss the application of NPT during complex intervention development to identify barriers and facilitators to implementation of the STAMINA lifestyle intervention (Additional File 1) in the NHS.

## Methods

### Study context, design and approvals

**Fig. 1:**
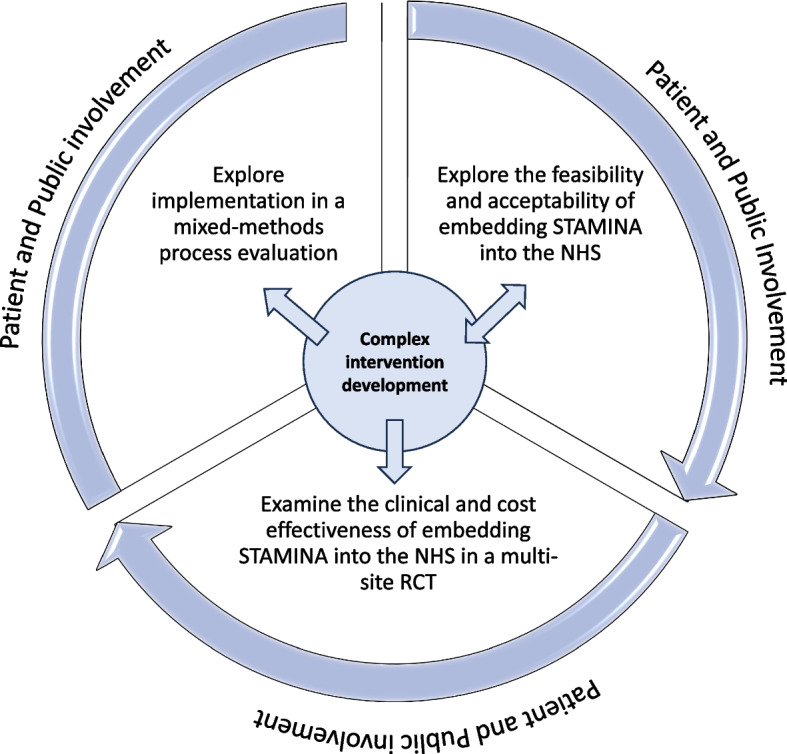
Overview of the STAMINA programme grant for applied research

This study contributes to a National Institute for Health and Care Research (NIHR) funded Programme Grant for Applied Research (PGfAR) aiming to evaluate the clinical and cost-effectiveness of embedding a tailored lifestyle intervention for men with prostate cancer on ADT into NHS prostate cancer care with delivery partner Nuffield Health (ISRCTN: 46385239) (See Fig. 1). The STAMINA lifestyle intervention is a behaviourally informed supervised exercise programme with dietary information endorsed by NHS healthcare professionals and delivered by upskilled community-based personal trainers. The STAMINA complex intervention was developed and refined in accordance with the Medical Research Council (MRC) guidance for the development of complex interventions [14]. To date, we have published work on the early phases of intervention development which were exploratory in nature and led to the definition of target behaviours and initial drafts of intervention materials [12, 13]. In this paper we provide a detailed account of two stakeholder workshops underpinned by NPT to explore future implementation during the later stages of complex intervention development.

Stakeholders can help to identify priorities, understand contextual problems and help find solutions that may make a difference to future implementation of interventions in the real world [14]. There are multiple methods for capturing stakeholder’s expert opinion, e.g., interviews, questionnaires, workshops [15]. Workshops provide a time-efficient method for collecting rich qualitative data from stakeholder groups and provide an opportunity to explore similarities and differences in opinion between and within professional and patient groups. Moreover, workshops enable stakeholders to be part of the decision-making during intervention development which is important for facilitating engagement and future implementation of an intervention or health problem [14]. This methodology has been used in previous studies developing complex interventions in the context of diabetes [16] and digital health [17].

**Fig. 2:**
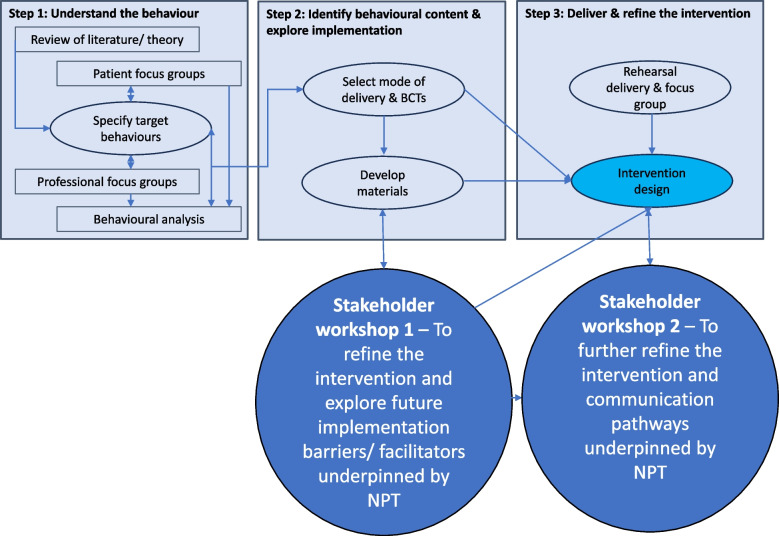
Contribution of two stakeholder workshops to the development and refinement of the STAMINA complex intervention

Stakeholder workshop 1 (SW1) was held in February 2019 building on the previously published review of evidence and qualitative exploration of barriers and facilitators to the patient and professional target behaviours (Table 1). Stakeholder workshop 2 (SW2) was held in January 2020 ahead of the complex intervention being finalised and evaluated in a pragmatic randomised controlled trial (RCT) (See Fig. 2).

**Table 1:** Specification of patient and professional target behaviours

Action	Actor	Context	Target	Time
Recognise patients suitable for exercise	Consultant or key worker	NHS prostate cancer care pathway, secondary care	Men with prostate cancer on Androgen Deprivation Therapy	At diagnosis, treatment or follow-up
Recommend exercise training	Consultant or key worker	NHS prostate cancer care pathway, secondary care	Men with prostate cancer on Androgen Deprivation Therapy	At diagnosis, treatment or follow-up
Refer for exercise training	Keyworker	NHS prostate cancer care pathway, secondary care	Men with prostate cancer on Androgen Deprivation Therapy	At diagnosis, treatment or follow-up
Support patients first contact with community gym provider	Fitness manager	Nuffield Health	Men with prostate cancer on Androgen Deprivation Therapy	On receipt of referral
Conduct and record results of a fitness test	Exercise physiologist or personal trainer	Nuffield Health	Men with prostate cancer on Androgen Deprivation Therapy	At baseline, 3-, 6- and 12-months
Tailor the exercise prescription	Exercise physiologist or personal trainer	Nuffield Health	Men with prostate cancer on Androgen Deprivation Therapy	At baseline and reviewed at 3-, 6- and 9-months
Deliver supervised exercise	Personal trainer	Nuffield Health	Men with prostate cancer on Androgen Deprivation Therapy	Twice a week for 12 weeks then once per month (maximum) for the remaining 9 months
Provide behavioural support	Fitness manager, exercise physiologist or personal trainer	Nuffield Health	Men with prostate cancer on Androgen Deprivation Therapy	At every supervised session and progress review
Compile progress reports	Exercise physiologist or personal trainer	Nuffield Health	Men with prostate cancer on Androgen Deprivation Therapy	At 3-, 6- and 12-month follow-up
Exercise twice per week	Men with prostate cancer on Androgen Deprivation Therapy	Nuffield Health	NA	For 12 months

Regulatory and ethical approvals were sought prior to the commencement of research activities from Sheffield Hallam University (Reference: ER10748795) and the NHS (REC reference: 18/NW/0738 / IRAS project ID: 254343). Written informed consent was collected from all participants prior to research activity.

#### Participants

Each workshop brought together representatives from key stakeholder groups purposively sampled to include NHS healthcare professionals (HCPs) working in prostate cancer care, community-based exercise professionals (EPs), people with advanced prostate cancer (patients) and their families/ carers, behaviour change experts and researchers. Stakeholders were invited by email or face-to-face to a 1-day workshop. A different group of stakeholders were invited to each workshop to capture a large range of knowledge, experience and opinion and to prevent possible time burden associated with attendance. Some of the participants were known to the research team due to their involvement with STAMINA in earlier work packages.

#### Materials

Two semi-structured topic guides underpinned by NPT [8] were developed to guide stakeholder workshop 1 and 2 (Additional file 2). An additional question related to the content, format and structure of intervention materials was added to support ongoing parallel intervention development.

Workshop manuals were designed to facilitate networking and discussion during the stakeholder workshops. The manuals contained: a summary of the STAMINA programme, speaker biographies, list of attendees, a copy of the slide deck presented including example training and intervention materials, space to record notes and references for further reading. At the end of the workshop participants were provided with a feedback form to capture further feedback, related to the STAMINA Lifestyle Intervention, the STAMINA programme and the delivery of the workshop.

Participants were not provided with any materials before or after the workshop.

#### Procedure

Stakeholder workshops were delivered in a conference room in Sheffield. Workshop participants were allocated a group table comprised of representatives from all stakeholder groups for facilitated cross-group discussion [18] considering impacts across these groups. The format of both workshops included an introduction to the table facilitator and full disclosure of the workshop aims followed by short explanatory presentations on various intervention elements by STAMINA research team members and task-based table discussions led by a facilitator (SR, RRT, ES, LS, ST, LB) and broad topic guide (Additional File 2) [19]. Each table discussion was followed by a facilitated (DM) whole group discussion as an opportunity for debate, sense checking and conclusion generation. Table and group facilitators were members of the research team including both male and female Research Assistants, Research Fellow, Senior Lecturer, Qualitative Expert, and Professors with the following credentials (MSc, PhD, MD). All facilitators had a minimum 1 year of facilitation experience and were guided by a qualitative research expert (ES). There were no other people in attendance of the workshop, and nobody withdrew from the study.

Notes from the table and whole group discussions were written individually by table facilitators in workbooks and on flip-chart paper (i.e., discussions were not audio recorded or transcribed verbatim). Stakeholders were provided with feedback forms to complete at the end of the session. Moreover, written feedback was collated by table facilitators and the research team to produce a summary that was sent to all stakeholders who were then invited to send any further comments to the team via email.

#### Analysis

An inductive and deductive approach to thematic analysis was guided by Braun and Clark’s six phases to thematic analysis [20]. Researchers familiarised themselves with the data whilst transcribing feedback during the workshop and re-reading whilst collating data to be sent to stakeholders for further comment. NPT constructs and sub-constructs provided a framework for analysis of the stakeholder workshop discussions and feedback forms with coding identifying what type of stakeholder provided the information and on which component of the intervention they were commenting on allowing us to pay attention to uncertainties across the different stakeholder groups and specific elements of the intervention [21]. Where data was not deemed relevant to the NPT constructs/sub-constructs then this was coded inductively by identifying patterns of potential interest. Codes were then collated with related codes to form groups ( i.e., new themes) which were discussed, refined and named where consensus was achieved between researchers during an iterative process of analysis (Table 2). Data was managed using Microsoft Office.

**Table 2:** Normalisation process theory constructs and sub-constructs for the STAMINA programme

Using Normalisation Process Theory in developing/refining the STAMINA intervention	
**Core construct**	**Sub-constructs**
1. Coherence—*Sense-making work that people do individually or collectively: understanding the purpose, value, and benefits of the STAMINA programme*	1.1 Differentiation: how is STAMINA different from other interventions 1.2 Communal Specification: building a shared understanding of aims, objectives, benefits of STAMINA 1.3 Individual Specification: understanding specific tasks and responsibilities within STAMINA 1.4 Internalization: understanding the value, benefits, importance of STAMINA
2. Cognitive Participation—*Relational work that people do to build and sustain a community of practice: getting buy-in, STAMINA champions*	2.1 Initiation: whether key participants are working to drive STAMINA implementation forward 2.2 Enrolment: strategies used to engage (buy-in), sustain engagement, and help secure implementation 2.3 Legitimation: ensuring participants believe it is right for them to be involved and they can make valid contribution 2.4 Activation: collectively defining actions and procedures needed to sustain STAMINA
3. Collective Action—*Operational work that people do to enact a set of practices:* *how STAMINA works in day-to-day practice (including roles/resources), communication pathways*	3.1 Interactional Workability: interactional work people do when operationalizing STAMINA 3.2 Relational Integration: knowledge work to build accountability and maintain confidence in STAMINA 3.3 Skill set workability: the allocation work that underpins the division of labour built up around STAMINA 3.4 Contextual integration: managing STAMINA through allocation of resources, execution of protocols, policies, procedures
4. Reflexive monitoring—*Appraisal work people do to assess and understand the ways a new set of practices affect them and the others around them: processes for measuring outcomes (team and individual) and feeding back to those delivering (HCPs, EPs) or taking part (patients) in STAMINA*	4.1 Systemization: collecting information to determine the effectiveness/utility of STAMINA 4.2 Communal appraisal: participants working together to evaluate STAMINA 4.3 Individual Appraisal: participants working experientially as individuals to appraise its effects on them and the contexts in which they are set 4.4 Reconfiguration: redefining procedures or modifying practices within STAMINA

The analysis was led by a female Research Fellow (PhD) with expertise in qualitative research and application of the NPT (ES). Data was cross-checked by two independent female researchers, one a Research Fellow (SR) the other a Research Assistant (RRT) with a minimum of three years’ experience analysing qualitative data and the following credentials, BSc, MSc, PhD. Inconsistencies were resolved through discussion.

## Results

### Stakeholder Workshop 1

Twenty-eight stakeholders attended SW1 (Table 3). Workshop participants discussed the content of the intervention and associated training packages, with a view to its potential integration into routine cancer care. We explored stakeholders’ perceptions of STAMINA across the four NPT constructs and sub-constructs to help understand perceived potential benefits of STAMINA for men with prostate cancer, in addition to factors impacting upon its future implementation (Additional File 3).

**Table 3:** Stakeholder workshop attendees

a: Stakeholder workshop 1 attendees	
**Stakeholder**	**Number of attendees**
Patient and Public Involvement members	8
Nuffield Health senior staff	3
Nuffield Health Exercise Professionals	6
Academic and community Exercise Professionals	2
Health Economist	1
Health Psychologist	1
CCG	3
Cancer alliance	1
Healthcare Professionals (secondary care)	3
**Total Number**	28
b: Stakeholder workshop 2 attendees	
**Stakeholder**	**Number of attendees**
Patient and Public Involvement members	4
Nuffield Health senior staff	3
Nuffield Health Exercise Professionals	1
Academic and community Exercise Professionals	2
Health Psychologist	1
CCG	1
Healthcare Professional (secondary care)	9
Research Nurse (secondary care)	1
Healthcare Professional (primary care)	3
Macmillan rehab lead	1
PCUK	2
**Total Number**	**28**

#### Coherence: understanding the purpose, value, and benefits of the STAMINA programme

Within NPT the embedding of a practice (normalization) is made possible by “a set of ideas about its meaning, uses, and utility; and by socially defined and organized competencies” [8] (p542) which hold the practice together. Stakeholders in workshop one recognised the importance of highlighting STAMINA’s unique selling points in professional training and patient-facing materials—including how the intervention is tailored to address individual needs (differentiation). Further key factors were ensuring that all stakeholders were made aware of the evidence base in support of the intervention (communal specification); that HCPs and EPs understood their individual roles and responsibilities within STAMINA (individual specification); and that patient-facing materials (such as STAMINA information booklets) should highlight the supportive evidence base, illustrated by patient stories, pictures and quotes. Together these factors were seen to make the practice “meaningful”, for example increasing HCP’s confidence in referring patients to the STAMINA Lifestyle Intervention (Additional file 3).

#### Cognitive participation: getting buy-in, STAMINA champions

This construct explores the “symbolic and real enrolments and engagements of human actors” [8] (p 243) that enable them to work together to build and sustain a community of practice. It was important to explore participants opinions on the possibility for the introduction of STAMINA in routine prostate cancer care – would HCPs and EPs be prepared to invest time and resource into getting STAMINA up and running [11], and would men with prostate cancer on ADT be keen to participate? To facilitate HCP and EP buy-in, stakeholders highlighted the importance of enrolling STAMINA “champions” located in both the NHS and Nuffield Health to engender confidence in the intervention (enrolment) and that training would need to be flexible with advanced notice to fit in with existing roles and commitments (initiation, enrolment). It was also highlighted that demonstrating the expertise of Nuffield Health in delivering exercise programmes to clinical populations (e.g., cystic fibrosis, joint pain) and their charitable status would be key to gaining professional trust in the intervention (legitimation). For patients, clear information about the benefits of exercise whilst undergoing ADT were emphasised as being important for patient buy-in with information presented by professionals and in booklet format (enrolment) (Additional file 3).

#### Collective Action: how STAMINA works in day-to-day practice (including roles/resources), communication pathways

This construct considers how actors work collectively towards specific goals as part of a particular practice [8]. Discussions focussed largely on early stages of implementation, for example, enabling HCPs and EPs to complete related training packages by offering different modes of deliveries (e.g., face-to-face versus remote) and hosting them on internal systems (interactional workability, contextual integration). Participants in workshop one also identified a need for an established referral and communication pathway, particularly in relation to assessing patient eligibility for participation and clear processes for raising concerns (relational integration; contextual integration). Furthermore, there were discussions about the current and required skillset of different roles within the fitness and wellbeing industry (e.g., physiologists, physiotherapists, and personal trainers) to identify the most suitable role and subsequent training needs to deliver the STAMINA programme – with a particular focus on having the skills and confidence to tailor exercise for men with prostate cancer who often have a number of health comorbidities (skill set workability).

Similarly, from the patient perspective, it was considered important to have a trained exercise professional to deliver the intervention and access to a medical professional for clinical support/ query resolution where required (skill set workability). Moreover, patients believed that the programme would work best if a variety of one-to-one and group sessions were offered (Contextual integration) (Additional file 3).

#### Reflexive monitoring: processes for measuring outcomes (team and individual) and feeding back to those delivering (HCPs, EPs) or taking part (patients) in STAMINA

The final NPT core construct considers how actors work together to continuously evaluate the outcomes of a practice, and in turn how these impact on its potential for normalization [8]. Discussions briefly touched on developing clear systems for clinical escalations in emergency scenarios (systemization); as well as methods of assessing the professional training packages (communal appraisal, individual appraisal); and adapting training materials and intervention documents (i.e., progress reports) to fit with existing technological systems (reconfiguration). Factors related to this construct were discussed more extensively in SW2 (Additional file 3).

#### Intervention refinement following Stakeholder Workshop 1

Following feedback from stakeholders the STAMINA complex intervention was refined to optimise the process of embedding supervised exercise into the prostate cancer care pathway ahead of a pragmatic RCT [8]. Key changes included hosting high level training on Nuffield Health internal systems and mandating completion by all front of house staff, management, and sales teams to enhance awareness related to operational procedures. Moreover, the decision was made to upskill personal trainers to deliver and support patient exercise behaviour as their role within Nuffield Health more closely aligns with the requirements of delivering SLI compared to that of a physiotherapist or physiologist. Information about Nuffield Health (e.g., charitable status, previous involvement in clinical research and exercise delivery, skillset, and mandatory training) was also added to the HCP training package and patient-facing materials alongside case study examples (quotes, patient stories, pictures etc.) to enhance understanding of the intervention purpose and subsequent increase buy-in.

### Stakeholder Workshop 2

Twenty-eight different stakeholders attended SW2 i.e., they had not participated in SW1 (Table 3). We built on the feedback from SW1 to frame discussions on the refined intervention. Discussions focussed on further refinement; progress reporting; communication pathways; and involved detailed consideration of the integration of STAMINA in day-to-day practice in both the NHS and charitable sector. There were some brief discussions related to the NPT construct of Coherence, where participants in workshop two re-emphasised the importance of STAMINA’s grounding in a supportive evidence-base and tailoring to fit individual patient’s needs to facilitate normalization (differentiation). Factors related to the construct of Cognitive Participation involved consideration of how the format of the patient facing materials might be refined to enhance acceptability of their use in the gym and prevent unwanted attention from the general public. It was also agreed that information about suitable clothing should be added so that men do not feel the need to invest in expensive kit (enrolment, legitimation) (Additional file 3).

#### Collective Action: how STAMINA works in day-to-day practice (including roles/resources), communication pathways

To implement STAMINA, key stakeholders need to work together to achieve common goals. Factors related to the construct Collective action were therefore discussed, including consideration of how HCPs and EPs might work together in the future to communicate key outcomes e.g., sharing patient progress with primary care staff (interactional workability). Specific roles and responsibilities in operationalizing the intervention were explored in detail, with a particular emphasis on streamlining progress reporting systems. Stakeholders also emphasised the role of the patient in taking ownership for recording their progress (skill set workability). The sub-construct of contextual integration focuses on how the introduction of a new practice affects “the mechanisms that link work to existing structures and procedures, and for realizing material and symbolic resources for them” [8] (p545). HCP and EP attendees highlighted the importance of introducing communication pathways and reporting systems that could integrate within existing systems and that they could easily incorporate within their day-to-day practice, and that these should be clearly set out in initial training packages (contextual integration). This included systems for reporting safety/health issues such as Nuffield Health’s internal Clinical Escalation policy and team (Additional file 3).

#### Reflexive monitoring: processes for measuring outcomes (team and individual) and feeding back to those delivering (HCPs, CEPs) or taking part (patients) in STAMINA

SW2 involved detailed discussion of factors related to the refinement of systems for collecting information to determine the utility/effectiveness of STAMINA (Additional File 3). There was lengthy consideration of the format, function, language/terminology, and purpose of the progress report e.g., that it should not act as safety reporting tool. Similarly, participants in workshop two proposed and agreed that the STAMINA diary should act as a self-monitoring tool to support patient behaviour change and maintenance and should be separate to the logbooks designed for data capture/ research purposes only (systemization). Leading on from this, stakeholders discussed how patients and EPs should use the report as a tool to aid discussions to evaluate progress (communal appraisal); and that patients should be provided with the opportunity to voice opinions on their personal progress e.g., within the STAMINA diary (individual appraisal). The latter factor linked to the NPT sub-construct of legitimation, in giving men confidence to participate. Possible enhancements to the patient facing materials were also discussed e.g., adding patient stories and photographs (reconfiguration).

#### Intervention refinement following Stakeholder Workshop 2

Following SW2, the patient facing materials were refined to increase acceptability based on stakeholder recommendations. More specifically, i) the STAMINA diary was reduced from A4 to A5 and wire bound to increase usability, ii) all STAMINA logos were removed from the front and back cover and replaced with standard Nuffield Health branding to reduce the likelihood of patients ‘standing out’ and receiving subsequent unwanted special attention and iii) additional information was added to depict appropriate clothing for exercise to enhance inclusivity. Moreover, an email communication pathway, embedded into current internal systems and IT infrastructure was established for reporting/escalating problems and discussing patient progress between Nuffield Health and the NHS. The method was chosen for its simplicity, safety (e.g., end-to-end encryption) and future proofing (e.g., likely to work in the future regardless of changes made to internal IT systems) (Additional file 3).

## Discussion

This paper describes the application of NPT to complex intervention development for management of ADT side-effects in prostate cancer. Behaviour change theories [22, 23] guided the early intervention development stages which were later complemented by Implementation Science, i.e., NPT [8]. Feedback was captured from 56 stakeholders during two stakeholder workshops to enhance possibilities for normalization, as they “play an important part in feeding back into notions of the coherence and meaningfulness of a practice” [8]. Discussions and analysis centred on NPT led to further development and refinement of the STAMINA complex lifestyle intervention including patient facing materials and professional training packages. Moreover, consideration for individual roles and infrastructure within the specified contexts led to the identification of a clear and simple communication pathway to fit within the organisational structures of the NHS and delivery partner, Nuffield Health.

At present, the provision of supervised exercise into the prostate cancer care pathway, as recommended by NICE, is rarely offered due to a multitude of barriers related to implementation [4]. As described previously [24], NPT was used to identify barriers and facilitators related to practicalities of NHS delivery in a sequential fashion across the two workshops. In the early phases of intervention development (i.e., SW1), the constructs of Coherence and Cognitive Participation (and related sub-constructs) were of particular relevance in our analysis. All stakeholders identified ‘buy-in’ and a good understanding of the underpinning evidence in support of the intervention as a necessary precursor to its implementation. In particular, the introduction of STAMINA Champions, information about the charity sector and clarity on professional roles were collectively identified as ways to increase HCP confidence to recommend and refer patients for exercise. Over the past decade, the use of champions has become a focal point of implementation research with systematic reviews evidencing champions as one of the core determinants of implementation success in healthcare settings [25]. However, less is known about the use of champions in other contexts. In the present study, EPs agreed champions could be beneficial in the charity sector though greater emphasis was placed on which professionals should be trained to deliver the intervention and where the training was hosted as a facilitator to enhance early ‘buy-in’. These differences highlight the importance of learning about the target contexts so that implementation strategies can be tailored accordingly (i.e., it is not a one size fits all).

The NPT constructs Collective Action and Reflexive Monitoring (plus sub-constructs) were the main discussion points of SW2. Discussions were centred on how to operationalise SLI into routine practice with consideration for future refinements ahead of delivery in the ever-changing context of the NHS and charity sector (Reconfiguration). For example, a simple and clear communication and reporting pathway between HCPs, EPs, and patients was favoured against a more complex reporting system that may require specialist software/ resource and may not be accessible to all contexts involved, now or in the future. Moreover, stakeholders agreed that intervention and research related information should be presented in separate materials to increase internal validity and so that minimal, if any, changes are required to the intervention materials ahead of national scale implementation (if found to be effective) – this is advantageous from both a resource and time perspective. Failure to address context specific changes could constitute a major barrier to appraising the transferability and applicability of findings following our planned RCT and may contribute to furthering the gap between research, policy, and practice [26]. Instead, the workshops developed our understanding of the local contexts so that a modifiable intervention could be developed and evaluated in our process evaluation to enable future implementation in a timely fashion (if found to be effective).

### Strengths & Limitations

Consideration of factors related to implementation as part of an iterative intervention development process was deemed essential for intervention optimisation ahead of testing in the context of a RCT. This blended approach is strengthened by its methodological rigour, time efficiency and likeliness of success compared to more traditional and sequential methods which tend to retrospectively explore implementation following intervention development and efficacy trials. Subsequently, the STAMINA Lifestyle Intervention was found to be feasible and acceptable, and delivered with moderate to high fidelity, when embedded into the NHS and Nuffield Health during a feasibility study [27].

More specifically, delivering two stakeholder workshops at different phases of intervention development enabled the research team to employ the four constructs of NPT to sequentially explore and identify determinants of (potential) future implementation. Furthermore, offering two workshops enhanced stakeholder reach thus providing valuable insight into patient perspective and organisational specific information which has been found to increase the adoption, implementation, and sustainability of interventions [28]. Subsequently, attendance at the workshops may have had additional implementation benefits beyond the primary aim focussing on the intervention. For example, workshop attendance may have acted to enhance Coherence and Cognitive Participation and support delivery of the trial as most clinical stakeholders submitted an expression of interest for their NHS Trust to be involved in embedding supervised exercise into the NHS as part of the STAMINA RCT.

Application of the NPT during intervention development enabled us to identify and characterise key barriers and facilitators of future implementation of the STAMINA Lifestyle Intervention and aid intervention development as part of an iterative process. However, in certain instances, application of the NPT was challenging as during the analysis phase some stakeholder feedback was mapped onto multiple constructs/sub-constructs demonstrating the multifaceted interactions of different elements of a complex intervention. For example, discussions around the format and purpose of the patient diary spanned the sub-constructs of legitimation and individual appraisal. Furthermore, the researchers who have a background in behaviour change had a natural desire to code discussion items at the individual level rather than more social processes. This may reflect the researcher’s familiarity with behaviour change framework s and/ or highlight that the TDF is another tool that can be used to assess implementation [22]. In this study NPT and TDF were used as complimentary to one another during intervention development and have the potential to improve how the interventions was designed through a robust, systematic method underpinned by multi-level theory as has been recommended by others [29]. We took an inductive and deductive approach to analysis and so both intervention and implementation barriers were explored in parallel to increase the usefulness and policy relevance of the research [30].

If the STAMINA Lifestyle Intervention is proven to be clinically and cost effective, future implementation work using NPT will be guided by the newly developed and published NPT coding manual – a tool created to provide a consistent and simple set of definitions of the core constructs of the theory to support researchers with qualitative analysis [31].

## Conclusions

In this paper, we discuss how the NPT was applied during complex intervention development and consider possible future implementation of embedding supervised exercise into routine clinical care. This blended approach demonstrates methodological rigour, time efficiency and increased likeliness of success compared to more traditional and sequential methods which tend to retrospectively explore implementation following intervention development and efficacy trials. All four constructs of the NPT were explored to identify context specific barriers and facilitators to future embedding of new pathways of care into the NHS with delivery partner Nuffield Health. The NPT will provide the explanatory framework to help understand how the STAMINA complex intervention is implemented, embedded, and integrated into routine practice in a planned definitive RCT (Trial registration: ISRCTN: 46,385,239).

### Supplementary Information

Additional file 1. TIDIeR Framework of the STAMINA Lifestyle Intervention. This file presents a description of the STAMINA Lifestyle Intervention in accordance with the TIDIeR framework.

Additional file 2. Topic guide. This file contains the topic guide that guided round table discussions at stakeholder workshop 1 and 2. The topic guide is based on the Normalisation Process Theory.

Additional file 3. Stakeholder discussions mapped onto the NPT. This file contains feedback from stakeholders, in stakeholder workshop 1 and 2, mapped onto the Normalisation Process Theory framework.

## Data Availability

All data generated or analysed during this study are included in this published article [and its supplementary information files].

## Abbreviations

EP: Exercise Professional
HCP: Healthcare Professional
MRC: Medical Research Council
NHS: National Health Service
NICE: National Institute for Health and Care Excellence
NIHR: National Institute for Health and Care Research
NPT: Normalisation Process Theory
PGfAR: Programme Grant for Applied Research
RCT: Randomised Controlled Trial
STAMINA: Supported exercise TrAining for Men wIth prostate caNcer on Androgen deprivation therapy
SW: Stakeholder Workshop
TDF: Theoretical Domains Framework
