# Supplementary material for: Using Normalisation Process Theory to explore the contribution of stakeholder workshops to the development and refinement of a complex behavioural intervention: the STAMINA lifestyle intervention
Source: Implement Sci Commun. 2024 Sep 2;5:94. doi: 10.1186/s43058-024-00629-1 (PMC11370076; doi:10.1186/s43058-024-00629-1)
Supplement: Supplementary file 1 — Additional file 1. TIDIeR Framework of the STAMINA Lifestyle Intervention. This file presents a description of the STAMINA Lifestyle Intervention in accordance with the TIDIeR framework. [file 43058_2024_629_MOESM1_ESM.docx]

**Additional File 1: TIDIeR Framework of the STAMINA Lifestyle Intervention**

| **Name** | Development and optimisation of the STAMINA Lifestyle Intervention (Supported exercise TrAining for Men with prostate caNcer on Androgen deprivation therapy) |
| --- | --- |
| **Why** | The National Institute for Health and Care Excellence (NICE) recommend that men on androgen deprivation therapy (ADT) for prostate cancer should be offered twice weekly aerobic and resistance exercise for 12 weeks to improve cancer specific fatigue and quality of life. However, these guidelines are not being delivered in the NHS highlighting the ongoing challenge of translating evidence into complex healthcare systems. |
| **What** | The STAMINA Lifestyle Intervention will include:   1. a recommendation to exercise from a healthcare professional from Urology or Oncology. 2. a referral to Nuffield Health. 3. an information pack and behaviourally informed diary to track progress. 4. an induction to the gym. 5. a tailored exercise programme. 6. twice a week supervised aerobic and resistance exercise for 12 weeks. 7. tapered supervised exercise between month 4 and 12. 8. quarterly progress reviews and behavioural support. |
| **Who provides** | The STAMINA Lifestyle Intervention will be offered to eligible participants as part of a pragmatic randomised controlled trial (RCT). Participants will be identified and recruited from one of twenty NHS trusts involved in delivering the RCT. Participants randomised to receive the intervention will be referred to Nuffield Health. Nuffield Health is the UK’s largest healthcare charity with a network of 37 hospitals, 114 fitness and wellbeing centres and workplace wellbeing facilities. |
| **How** | The STAMINA Lifestyle Intervention is designed to be delivered face-to-face one-to-one and face-to-face to small groups (maximum of 5 people). |
| **Where** | The STAMINA Lifestyle Intervention has been designed to be delivered at up to 20 Nuffield Health fitness and wellbeing centres as part of the RCT. More specifically, on the gym floor and in clinic/ consultation rooms. |
| **When and how much** | STAMINA Lifestyle Intervention participants will receive a 12-month exercise programme as part of the pragmatic RCT. In line with recommendations from NICE, participants will be offered supervised exercise biweekly for 12 weeks based on the patient and personal trainers’ availability. Supervision will be reduced for the remaining 9 months of the programme, but participants will be encouraged to continue exercising twice per week.  The full STAMINA exercise prescription includes:   - Frequency – twice per week - Intensity – moderate to hard - Type – aerobic and resistance - Time – 30-45 minutes aerobic and up to 4 sets, 8 – 12 reps resistance exercise.   In parallel, participants will be provided with a behaviourally informed STAMINA diary (for self-monitoring) and progress reviews including behavioural support underpinned by the Theoretical Domains Framework.   - Week 4 review – feedback and rewards - Week 6 review – feedback and goal setting - Week 12 review – feedback, social support, and habit formation - Week 26 review – feedback and monitoring - Week 39 review – feedback and goal setting - Week 52 review – feedback and action planning |
| **Tailoring** | The exercise programme will be tailored based on each participants capability, opportunity, and motivation to exercise. |
| **Modifications** | Modifications to the implementation of STAMINA ahead of the planned RCT are described in the main text of the manuscript. |
| **How well** | Intervention fidelity and acceptability will be assessed in the main RCT. |
