# Supplementary material for: Using Normalisation Process Theory to explore the contribution of stakeholder workshops to the development and refinement of a complex behavioural intervention: the STAMINA lifestyle intervention
Source: Implement Sci Commun. 2024 Sep 2;5:94. doi: 10.1186/s43058-024-00629-1 (PMC11370076; doi:10.1186/s43058-024-00629-1)
Supplement: Supplementary file 2 — Additional file 2. Topic guide. This file contains the topic guide that guided round table discussions at stakeholder workshop 1 and 2. The topic guide is based on the Normalisation Process Theory. [file 43058_2024_629_MOESM2_ESM.docx]

**Additional File 2: Stakeholder workshop topic guide based on the Normalisation Process Theory**

*A brief overview of the study will be given to the participants followed by general house-keeping rules and focus group etiquette. There will be an opportunity for the participants to ask questions before starting.*

- Opinions on the proposed content, format and structure of the intervention (HCP, patient and exercise professional elements and communication pathways)
- Does the intervention have a clear purpose for all participants (HCPs in the MDT, exercise professional and study patient participants)? (*COHERENCE*)
- How feasible is the delivery of the intervention (*COGNITIVE PARTICIPATION*, *COLLECTIVE ACTION*)
  - Opinions on the mode of delivery of intervention?
  - Opinions on the duration of intervention?
- Do participants believe the intervention will be put in place (*COGNITIVE PARTICIPATION, COLLECTIVE ACTION*)
  - What would be peoples’ motivations, barriers, capabilities to put in place?
  - Does intervention fit with individuals’ roles? Does the intervention fit with the overall organizational goals of the hospital team and the exercise professionals?
- Do participants believe the intervention will bring benefits (and be perceived as advantageous) for patients, for staff and for organisations? (*COHERENCE, REFLEXIVE MONITORING*)
  - How will benefits be recognised,
  - Ways to facilitate this

- How acceptable is the new HCP behaviour likely to be to patients (the ultimate recipients)? (*COGNITIVE PARTICIPATION*)
- How acceptable is the communication pathway likely to be to patients? (*COLLECTIVE ACTION*)

- Opinions on the proposed pathways of communication between health care professional and exercise professionals (*COLLECTIVE ACTION*)
  - What are the best ways of communicating between these two groups?
  - What is feasible?
  - How acceptable is this communication, to patients?
- How might the fidelity of the intervention be promoted? (*COLLECTIVE ACTION*)
- Will it be clear from the study what effects the intervention has had, will the team be aware of benefits? (*REFLEXIVE MONITORING*)
- Is there learning from related areas that could be helpful here e.g. cardiac rehabilitation. (*REFLEXIVE MONITORING*)
