# Supplementary material for: Using Normalisation Process Theory to explore the contribution of stakeholder workshops to the development and refinement of a complex behavioural intervention: the STAMINA lifestyle intervention
Source: Implement Sci Commun. 2024 Sep 2;5:94. doi: 10.1186/s43058-024-00629-1 (PMC11370076; doi:10.1186/s43058-024-00629-1)
Supplement: Supplementary file 3 — Additional file 3. Stakeholder discussions mapped onto the NPT. This file contains feedback from stakeholders, in stakeholder workshop 1 and 2, mapped onto the Normalisation Process Theory framework. [file 43058_2024_629_MOESM3_ESM.docx]

| **Stakeholder Workshop 1**  1. Coherence | |
| --- | --- |
| - 1. 1.1 Differentiation | Patient-facing and training materials should highlight STAMINA’s unique selling points (All, PFM)  Tailored interventions/tailored training (All) |
| - 1. 1.2 Communal Specification | Belief in the worth of STAMINA – e.g. the evidence base to support the intervention (All, PFM)  Understanding role of nutrition in pathway (All) |
| - 1. 1.3 Individual Specification | Understanding roles within STAMINA including referral/eligibility/exercise prescription/progress reporting (All)  Develop who is who schematic information sheet (CP) |
| - 1. 1.4 Internalization | PFM should include patient stories, quotes and pictures and include dietary advice/evidence to support (PI, PFM)  PFM and training should highlight evidence-base to support STAMINA (PI, HP, EP, PFM) |
| 2. Cognitive Participation | |
| 2.1 Initiation | Flexible training to fit with HCP and EP (HP, EP) |
| 2.2 Enrolment | STAMINA Champion in the NHS and NH would encourage buy-in (All)  Emphasise quality/experience and unique selling points of NH to build trust (PI)  PFM should provide clear information on ADT and exercise (PI, PFM)  Flexible training to fit with HCP and EP (HP, EP) |
| 2.3 Legitimation | EP present at HCP training or the use of videos of Eps explaining their role would be helpful in building trust (HP,EP)  Endorse/highlight quality of NH (including EP training/capabilities) to build trust (HP, EP)  PFM should highlight evidence-base to support STAMINA , Include patient stories in PFM/training (PI, PFM) |
| 2.4 Activation | Regional clinic managers as mentors for EPs – trouble-shooting activation issues (CP) |
| 3. Collective Action | |
| 3.1 Interactional Workability | Online training uploaded onto the Nuffield Academy website and include easy to follow videos (EP)  Training should consider conversations between HPs and patients regarding behaviour change and referral decisions (HP)  Example of good practice conversations (re exercise) with patients (HP)  Simplified pathway with focus on key lines of communication (CP) |
| 3.2 Relational Integration | Offer continuity of PTs for any given individual where possible (PI)  Set up of referral/communication pathways between EPs and HPs to maintain confidence in STAMINA (EP, HP, All)  Clear system for raising concerns – particularly in an emergency (CP)  Online patient area – feedback/tracking progress (CP) |
| 3.3 Skill set workability | Upskill personal trainers to facilitate the role of the physiologist in the STAMINA programme (EP)  Delivery of training should fit role/involvement with STAMINA (HP - content)  At least one consultant trained at each site (HP)  Medical professional at NH – for any queries (All)  Consider current skill set/responsibilities of EPs when identifying/enrolling EPs to work on STAMINA (EP, All) |
| 3.4 Contextual integration | Scheduling training, flexible delivery, range of delivery modes (in person, online) (HP, EP)  EP involvement to fit with existing on shift/off shift system, continuity of EPs where possible (EP)  Identifying adverse events/issues/problems, raising red flags (HP, EP, CP)  Offering a mix of 1to1/group sessions and venues (PI)  Compliance with data governance framework (CP)  Clarity of referral/appointment/reporting systems (CP) |
| 4. Reflexive monitoring: | |
| 4.1 Systemization | Clear, simplified communication pathway: from a clinical team champion (e.g. clinical nurse specialist) to a NH champion and clear system for raising concerns – particularly in an emergency (CP)  PFM to act as progress record (PI, PFM)  Individualised/site specific pathways of care and outcome data collection, utilising existing digital hub (CP) |
| 4.2 Communal appraisal | Open communication between HPs and EPs (CP) |
| 4.3 Individual Appraisal | Assessment of training – multiple choice/pop quiz (HP)  Progress reports/patient ownership (PI) |
| 4.4 Reconfiguration | Training materials uploaded to NH academy (EP)  Translating health care language to NH language (EP – content)  Data sent via digital hub (CP) |

| **Stakeholder Workshop 2**  1. Coherence: | |
| --- | --- |
| 1.1 Differentiation | Patient-facing and training materials should highlight STAMINA’s unique selling points (All, PFM)  Tailored interventions/tailored training (All) |
| - 1. 1.2 Communal Specification |  |
| - 1. 1.3 Individual Specification |  |
| - 1. 1.4 Internalization |  |
| 2. Cognitive Participation: Relational work that people do to build and sustain a community of practice | |
| 2.1 Initiation |  |
| 2.2 Enrolment  Strategies used to engage (buy-in),  sustain engagement and help secure implementation | Ensure the STAMINA diary is a convenient size/ format to use in the gym (PFM)  Ensure the STAMINA diary appearance does not make patients in the study stand out from other gym users (PFM)  Review dietary advice and focus on dietary recommendations whilst on ADT, e.g., diet swaps/ recipes (PFM)  Review exercise advice and add detail about the importance of following a tailored programme at all times (PFM)  Add examples/ recommendations of suitable clothing to wear in the gym (PFM)  Gain additional feedback on the amended PFM from the PPI with a particular focus on format and content (PFM, Gl)  Add information about the importance of social support for behaviour change, i.e., meeting others on the programme (Gl) |
| 2.3 Legitimation  Ensuring all participants believe it is right for them to be involved and they can make valid contribution | Give examples of clothes to wear to gym e.g. “Most men wear sweat pants or shorts and an old tee shirt” (PFM, GI) |
| 2.4 Activation  Collectively defining actions and  procedures needed to sustain STAMINA |  |
| 3. Collective Action: Operational work that people do to enact a set of practices | |
| 3.1 Interactional Workability Interactional work people do when operationalizing STAMINA | Explore using online platforms for the progress report to be communicated between CBEP and HCPs (CP)  Explore sharing patient progress with primary care as patients are often followed up in primary care (CP)  Review process of communicating patient information between key stakeholders and how this can be streamlined (CP)  Add contact details in all communication for clarity (Gl/CP) |
| 3.2 Relational Integration  Knowledge work to build accountability and maintain confidence in STAMINA |  |
| 3.3 Skill set workability  The allocation work that underpins the division of labour built up around STAMINA (To include patient role/responsibilities) | Recommendation for there to be two copies of the progress report for a) communication with HCPs and b) patient self-monitoring of behaviour (Gl)  Recommendation for the patient to take ownership in completing an independent progress review (Gl)  Recommendation for HCP to request and provide feedback to patient on exercise behaviour in follow-up clinics (Gl)  Remove HCP responsibility to confirm patient safety to continue with exercise (Gl)  Add detail to HCP professional training about how to use and complete the progress report in routine clinic appointment (Gl)  Recommendation to refer patients to see a dietician if they want to change their diet (Gl) |
| 3.4 Contextual integration  Managing STAMINA through allocation of resources, execution of protocols, policies, procedures | Recommendation to remove ’12 weeks’ from the title of the progress report and replace with space to add ‘date started’ and ‘date of review’ (Gl, CP)  Add detail to the HCP professional training detailing when progress reports will be received (Gl, CP)  Consider how the progress reporting system will work once the research components are removed (Gl, CP)  Keep the progress report as a paper document in line with PPI preference for completion (Gl, CP)  Provide patients with a cover letter in their patient booklet pack signposting them to information (PFM, Gl)  Provide patients with a self-monitoring tool (i.e., within the STAMINA diary) to share with their HCP in instances where HCPs do not receive or have time to review the progress reports (PFM, Gl)  Recommendation to create a communication pathway for reporting injuries or new/ changes in health that may influence delivery of supervised exercise (CP)  Provide additional protected time to CBEP to review progress at 6, 9 and 12 months (Gl) |
| 4. Reflexive monitoring: Appraisal work people do to assess and understand the ways a new set of practices affect them and the others around them | |
| 4.1 Systemization  Collecting information to determine the effectiveness/utility of STAMINA  (To include patients’ perceptions of efficacy/benefit)  **NOTE:** Some queries/comments also relevant to individual appraisal e.g. how a participant perceived symptoms etc – at 4.3 | **What data should be collected:**  Provide patients with a self-monitoring tool (i.e., within the STAMINA diary) to track progress related to side effects of treatment, exercise behaviour, other lifestyle changes and outcomes of exercising (PFM, Gl).  Remove specific details about the exercise programme from the progress report for HCPs (e.g., reps and sets) (Gl)  Remove section 1b from the progress report (i.e., the progress report is for HCP information only, no response from the HCP required) (Gl)  Add information to the progress report detailing the number of sessions attended, outcomes to date and any problems (i.e., what went well, what went less well, what might help in the future) (Gl) Provide patients with VAS and graphs to complete for visual progress (Gl)  **Terminology + tone of report:**  Consider re-naming the progress report due to personal interpretation of the word ‘progress’. Use the word participant instead of patient throughout all documents (PFM, Gl) Ensure the focus of the progress report is positive and about achievements (Gl)  **Purpose/how the report is used:**  Add detail to the HCP professional training to confirm that the progress report should be used as a guide for conversation only and does not act as a safety reporting tool (Gl)  **Content/purpose of the diary:**  Recommendation to separate information into a) PFM to support behaviour change and maintenance and b) logbooks for data capture/ research purposes only. PFM are personal and are to be completed/ kept by patients whereas logbooks should remain on site and be completed by CBEPs (PFM, Gl)  Move all information related to lifestyle change and NH to the PFM only (PFM, Gl)    Provide space in the PFM for patients to record independent exercise sessions (PFM)  Provide CBEP with copies of the PFM for reference (PFM, Gl)  Add detail to the CBEP training to advise CBEPs that it is their role to record data in the logbooks in line with protocol, to deliver tailored exercise in group supervised sessions, how many sessions will be delivered one-to-one and how it is determined when a patient is ready to be transitioned to group supervision (EP, Gl) |
| 4.2 Communal appraisal  Participants working together to  evaluate STAMINA | Ensure the patient and exercise professional have frequent discussions about patient progress and both have a way of maintaining their own records (Gl)  Recommendation to prevent patients from taking home logbooks that contain data required for the study (Gl)  Recommendation to provide patients with information about lifestyle change and behavioural support at frequent intervals during the programme to support patient progress (Gl) |
| 4.3 Individual Appraisal  Participants working experientally as individuals to appraise its effects on them and the contexts in which they are set | Provide patients with VAS and graphs to complete for visual progress in the STAMINA diary (PFM, Gl)  Ensure patients have their own tool to voice their opinion in instances where there is a disagreement with Nuffield Health (i.e., the STAMINA diary) (PFM, Gl)  Provide patients with their own diary which includes behavioural support and activities that mirror the topics of conversation and support provided by the CBEP. The aim is for the patient to complete their diary weekly (PFM, Gl) |
| 4.4 Reconfiguration  Redefining procedures or modifying practices | **Stakeholders suggested the following changes to content:**  Provide patients with VAS and graphs to complete for visual progress on goals personal to them (PFM, Gl)  Add space for patients to self-report how they felt in their supervised exercise session (PFM, Gl)  Ensure the STAMINA diary provides support for 12 months in line with the duration of the intervention (Gl)  Add patient stories and quotes to the STAMINA diary (PFM)  Add a contents page, contact details and instruction on how to use the diary at the beginning of the diary (PFM)  If the logbooks remain separated (i.e., week 1 -12 and week 13 – 52) add a summary page to the second booklet (Gl)  **General changes to patient materials suggested:**   - Add page numbers and consider text size - Some task boxes on the wrong page - goes onto second page - Add definitions of abbreviations - Text heavy- use more bullet points |
